# Filariasis Attenuates Anemia and Proinflammatory Responses Associated with Clinical Malaria: A Matched Prospective Study in Children and Young Adults

**DOI:** 10.1371/journal.pntd.0001890

**Published:** 2012-11-01

**Authors:** Housseini Dolo, Yaya I. Coulibaly, Benoit Dembele, Siaka Konate, Siaka Y. Coulibaly, Salif S. Doumbia, Abdallah A. Diallo, Lamine Soumaoro, Michel E. Coulibaly, Seidina A. S. Diakite, Aldiouma Guindo, Michael P. Fay, Simon Metenou, Thomas B. Nutman, Amy D. Klion

**Affiliations:** 1 Faculty of Medicine, Pharmacy and Dentistry, University of Bamako, Bamako, Mali; 2 Biostatistics Research Branch, National Institute of Allergy and Infectious Diseases, National Institutes of Health, Bethesda, Maryland, United States of America; 3 Laboratory of Parasitic Diseases, National Institute of Allergy and Infectious Diseases, National Institutes of Health, Bethesda, Maryland, United States of America; Ege University, Turkey

## Abstract

**Background:**

*Wuchereria bancrofti* (Wb) and *Mansonella perstans* (Mp) are blood-borne filarial parasites that are endemic in many countries of Africa, including Mali. The geographic distribution of Wb and Mp overlaps considerably with that of malaria, and coinfection is common. Although chronic filarial infection has been shown to alter immune responses to malaria parasites, its effect on clinical and immunologic responses in acute malaria is unknown.

**Methodology/Principal Findings:**

To address this question, 31 filaria-positive (FIL+) and 31 filaria-negative (FIL−) children and young adults, matched for age, gender and hemoglobin type, were followed prospectively through a malaria transmission season. Filarial infection was defined by the presence of Wb or Mp microfilariae on calibrated thick smears performed between 10 pm and 2 am and/or by the presence of circulating filarial antigen in serum. Clinical malaria was defined as axillary temperature ≥37.5°C or another symptom or sign compatible with malaria infection plus the presence of asexual malaria parasites on a thick blood smear. Although the incidence of clinical malaria, time to first episode, clinical signs and symptoms, and malaria parasitemia were comparable between the two groups, geometric mean hemoglobin levels were significantly decreased in FIL− subjects at the height of the transmission season compared to FIL+ subjects (11.4 g/dL vs. 12.5 g/dL, p<0.01). Plasma levels of IL-1ra, IP-10 and IL-8 were significantly decreased in FIL+ subjects at the time of presentation with clinical malaria (99, 2145 and 49 pg/ml, respectively as compared to 474, 5522 and 247 pg/ml in FIL− subjects).

**Conclusions/Significance:**

These data suggest that pre-existent filarial infection attenuates immune responses associated with severe malaria and protects against anemia, but has little effect on susceptibility to or severity of acute malaria infection. The apparent protective effect of filarial infection against anemia is intriguing and warrants further study in a larger cohort.

## Author Summary

In many regions of the world, including sub-Saharan Africa, concomitant infection with multiple parasites is common. In order to examine the effects of filariasis, a chronic helminth infection, on immune responses and clinical manifestations of acute malaria infection, the authors followed 31 filaria-infected (FIL+) and 31 filaria-uninfected (FIL–) individuals living in a malaria-endemic area of Mali through an entire malaria transmission season for the development of clinical malaria (fever or other symptoms of malaria in the setting of detectable blood parasites). Serum levels of inflammatory cytokines previously associated with severe malaria were decreased in FIL+ subjects at the time of acute clinical malaria. Although there were no differences between FIL+ and FIL– subjects with respect to the time of first episode of malaria or the number or severity of malaria episodes, filarial infection appeared to protect against the development of anemia during the malaria transmission season. These findings demonstrate that chronic filarial infection modulates the immune response to acute malaria. The apparent effect on anemia is intriguing and deserves further study.

## Introduction

Filarial infections and malaria are coendemic in many areas of the world, including sub-Saharan Africa, where human coinfection with malaria and filarial parasites is common [1], [2], [3]. Chronic filarial (helminth) infection is associated with skewing of parasite-specific immune responses towards a Th2/Treg cytokine pattern [4]. Furthermore, studies have demonstrated extension of this skewing of the immune response in helminth-infected individuals to bystander antigens with clinically relevant effects on responses to immunization with tetanus toxoid [5], [6] and oral cholera vaccine [7]. In contrast to tissue invasive helminth infection, malaria is characterized by an acute inflammatory response with increases in serum proinflammatory cytokine and chemokine levels, including IL-1β, IL-6, IL-8, TNFα, IP-10, IL-1ra and IL-12p70 [8], [9], [10], [11], [12], [13], [14]. Although several of these mediators have been implicated in control of the infection [9], [10], they are also associated with increased severity of clinical disease [11], [12], [13], [14]. Consequently, with the implementation of worldwide programs to eliminate filariasis and other helminth infections, questions have arisen regarding the impact of antifilarial chemotherapy (and de-worming) on immune responses to acute malaria infection and the potential effect of this immune modulation on malaria morbidity and mortality [15].

A number of studies have demonstrated that chronic filarial infection can suppress human immune responses to malarial antigens *in vitro*, including a recent study that showed an association of patent filarial infection with decreased malaria antigen-specific IL-12p70/IFNγ production [4]. This effect was reversed by the addition of neutralizing antibodies to IL-10. Furthermore, filaria-infected individuals with asymptomatic malaria parasitemia have been shown to have lower frequencies of malaria-specific Th1 and Th17 cells [16]. Despite clear evidence for chronic modulation of malaria-specific immune responses in vitro, few studies have addressed the effects of chronic filarial infection on immune responses and clinical outcomes in the setting of acute malaria in human populations.

The current study was designed to determine if pre-existent filarial infection alters the frequency and/or severity of clinical malaria in children and young adults in a coendemic area in Mali where malaria transmission follows a seasonal pattern. Cytokine profiles at the time of presentation with acute malaria were also compared between subjects with and without concomitant filarial infection.

## Methods

### Objectives

The study was designed as a matched prospective study of the effects of filarial infection on the incidence, severity and immune responses to malaria infection.

### Study site

The study was conducted in the villages of Tieneguebougou and Bougoudiana in the district of Kolokani, approximately 105 km northwest of Bamako, Mali, between June 2007 and December 2007. Prior studies in these villages had demonstrated a high prevalence of *Wuchereria bancrofti* (Wb) and *Mansonella perstans* (Mp) microfilaremia in the population, but no evidence of onchocerciasis. A single round of mass drug administration (MDA) with ivermectin and albendazole was conducted by the National Program to Eliminate Lymphatic Filariasis prior to the study in May 2006, and study subjects participated in a second round of MDA during the study in August 2007. Malaria transmission in this region is seasonal (June–December) with a cumulative EIR of 19.2 infective bites/person in a neighboring village in 2000 [17].

### Study participants

**Figure 1:**
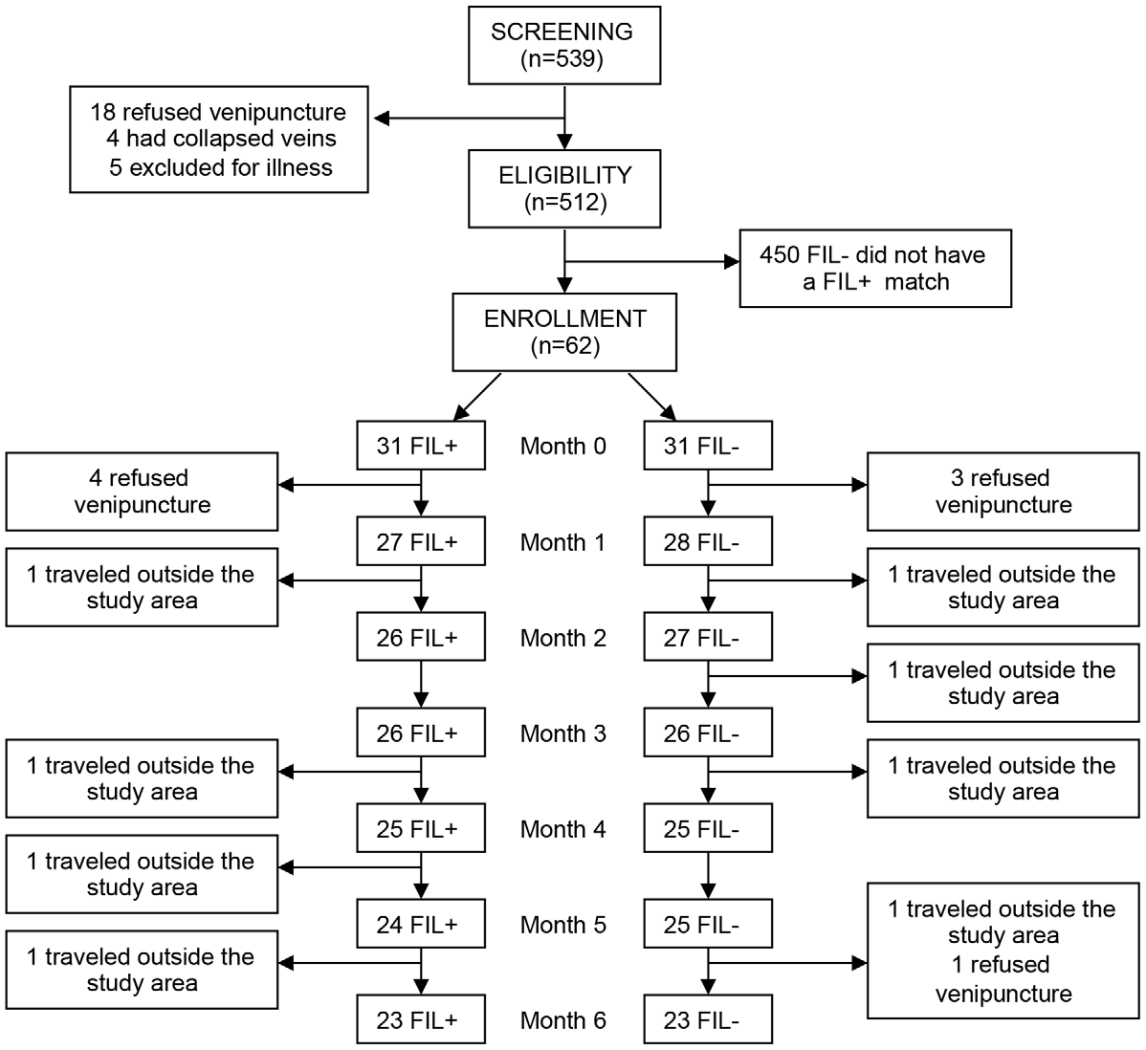
Study flowchart.

The study flowchart and selection of study participants is shown in Figure 1. Non-pregnant volunteers (n = 539) of both genders, aged from 1 to 20 years, were screened with a brief medical history and physical examination. Subjects with evidence of severe or chronic illness, a history of allergy to anthelmintic or antimalarial agents or plans to relocate out of the village during the study were excluded, as were subjects who refused venipuncture or had difficult venous access. The remaining subjects underwent laboratory testing, including pregnancy testing (if appropriate), hemoglobin (Hgb) measurement using a portable analyzer (Hemocue, Lake Forest, CA), Hgb typing by HPLC (D-10 instrument; Bio-Rad, http://www.bio-rad.com), daytime thick smear for detection of malaria and/or Mp infection, and circulating antigen (CAg) ELISA for Wb (TropBio, Townsville, Australia). Based on these results, potential participants were divided into two groups: 1) FIL+: individuals with confirmed active filarial infection with Wb and/or Mp, as defined by the presence of CAg and/or microfilaremia (mf) and 2) FIL−: individuals without evidence of active filarial infection (negative for CAg and mf). Thirty-one FIL+ subjects were identified and matched to FIL− subjects on the basis of gender, age (within 4 years), and Hgb type. Glucose-6-phosphate dehydrogenase (G6PD) deficiency was assessed by restriction enzyme analysis [18] in all 62 subjects. Filarial infection status was confirmed at the time of enrollment in the prospective study, and reassessed after 3 and 6 months by Nuclepore™ filtration of 1 ml of blood drawn between 10 pm and 2 am and CAg ELISA (TropBio).

### Study procedures

All participants were evaluated weekly throughout the malaria transmission season. At each visit, symptoms and signs of clinical malaria were assessed, and if present, blood was obtained from a finger stick for preparation of Giemsa-stained thick and thin blood smears for detection and speciation of malaria parasites and Hgb measurement. In addition, subjects were encouraged to present to the study physician for evaluation if they felt ill. Blood smears and Hgb determinations were also performed at the start of the study and monthly thereafter, regardless of the presence of clinical signs or symptoms. Reading of these monthly blood smears was deferred until the end of the study unless the subject was symptomatic at the time of the blood draw. To minimize the potential effects of concomitant helminth infection, all subjects were treated with single dose praziquantel (40 mg/kg) and albendazole (400 mg) at the start of the study. Albendazole treatment was repeated every two months during the study period.

Clinical malaria was defined as the presence of fever (axillary temperature ≥37.5°C) or another symptom or sign compatible with malaria infection plus the presence of asexual malaria parasites on a thick blood smear. Malaria parasitemia was determined by counting the number of asexual *P. falciparum* parasites on Giemsa stained thick blood films until 300 leukocytes were observed. All slides were read by two independent microscopists, and differences of >10% were resolved by an expert microscopist. Parasite densities were calculated by multiplying the mean value for the two readers by 25 to give parasites/microliter.

Cases of uncomplicated malaria were treated with standard recommended doses of artesunate-amodiaquine (4 mg/kg artesunate and 10 mg/kg amodiaquine per day for 3 days). Cases of therapeutic failure were treated with quinine (10 mg/kg/day for 3 days). On days 1, 2, 3, 7 and 14 following treatment, subjects were evaluated for clinical signs and symptoms, and malaria smears and Hgb measurements were performed. Plasma levels of interferon (IFN)γ, interleukin (IL)-1α, IL-1β, IL-1ra, IL-6, IL-8, IL-10, IL-12p70, IP-10, and tumor necrosis factor (TNF)α were measured in plasma samples obtained prior to treatment by suspension array in multiplex (Millipore Corp, St. Charles, MO), according to the manufacturer's instructions. The limits of detection of the assay are 0.5 pg/ml for IFNγ, 0.63 pg/ml for IL-1α, 0.19 pg/ml for IL-1β, 10.97 pg/ml for IL-1ra, 0.79 pg/ml for IL-6, 0.32 pg/ml for IL-8, 0.41 pg/ml for IL-10, 0.23 pg/ml for IL-12p70, 1.14 pg/ml for IP-10, and 0.22 pg/ml for TNFα.

### Ethics

The study (NCT00471666) was approved by the ethical review committees of the Faculty of Medicine, Pharmacy, and Dentistry at the University of Bamako (Mali) and of the National Institutes of Allergy and Infectious Diseases (Bethesda, Maryland). Community permission for the study was obtained from village elders, and individual oral or written informed consent (for subjects ≥18 years of age) or parental informed consent and assent (for subjects <18 years of age) was obtained from all participants in French or Bambara, the local language. Oral consent was obtained for illiterate participants and parents of participants as approved by the ethical review committees and was documented by the signatures of a member of the study team and a witness.

### Statistical analysis

The ratio of the rates of clinical malaria events was calculated by Poisson regression adjusting for the time at risk [19]. Person-season was defined as the sum of the days at risk for all subjects during the first season divided by 168 days (24 weeks). The time to first episode of clinical malaria analysis used the Kaplan-Meier estimates and Cox proportional hazards [20]. Differences between the two groups were tested using the Mann-Whitney test (continuous responses) or Fisher's exact test (binary responses). Confidence intervals (CI) on the ratio of geometric means (GM) were calculated using a t-test on the log-transformed values and CIs on the difference in arithmetic means (for temperature) used t-tests on untransformed values. Wilcoxon signed rank test was used to test for changes over time and Spearman rank for assessment of correlation. Analyses were done in R [20] or GraphPad Prism (V5.0).

## Results

### Demographic and parasitologic characteristics of the study population

Baseline characteristics for the two groups are shown in Table 1. Overall, the subjects had a median age of 13 years, 65% were male, and the majority had normal Hgb (HgbAA). G6PD deficiency was present in 6/62 (14%) subjects and was equally common in the two groups. Baseline GM Hgb was similar between subjects with and without filariasis (12 g/dL in both groups). Malaria parasites were detected in a high proportion of subjects at baseline in both groups (58.1% and 64.5% in FIL+ and FIL−, respectively), but among those with detectable malaria parasites, parasitemia was low (GM 233 parasites/µl in FIL+ and 147/µl in FIL−, respectively; p = 0.06), with a level >1000 malaria parasites/µl in only one subject in the FIL− group. No intestinal helminth infections were detected in any of the subjects by stool examination, although intestinal protozoa (Giardia and Entamoeba) were found in 4 and 11 subjects, respectively.

**Table 1:** Baseline characteristics of the study population.

	Filaria-positive (n = 31)	Filaria-negative (n = 31)
Median age (range)*	13 (4–18)^A^	13 (4–20)^A^
Gender (M/F)*	20/11^A^	20/11^A^
Hgb status (AA/AC/AS)*	27/2/2^A^	27/2/2^A^
G6PD deficiency (%)	5/31(16.1%)^A^	4/31(12.9%)^A^
GM Hgb (range)	11.5 (7.8–15.5)^B^	11.3 (9.3–13.4)^B^
Malaria smear positive (%)	18/31 (58.1%)^B^	20/31(64.5%)^B^
Wb CAg positive (%)	11/31(35.5%)^C^	NA
Wb mf positive (%)	2/31 (6.5%)^C^	NA
Mp mf positive (%)	20/31(64.5%)^C^	NA

* matched by design.

A = from screening data.

B = from baseline data.

C = at either screening or baseline.

NA, not applicable.

Filarial status was assessed at baseline and at 3 and 6 months. Among the 31 FIL+ subjects, 11 (35.5%) were infected with Wb and 20 (64.5%) with Mp. Two subjects had detectable Wb microfilariae in the peripheral blood. During the course of the study, 3 FIL+ subjects, all of whom were Mp-infected, became FIL− (Table 2). As expected, the two subjects with Wb microfilaremia cleared their circulating mf at 3 months (after participating in the mass drug administration of ivermectin and albendazole in their village). Eleven FIL− subjects acquired filarial infection during the study: 7 became positive for CAg, 3 for Mp mf and 1 for both CAg and Mp mf.

**Table 2:** Changes in filarial status over the course of the study.

Nature of change	Filaria-positive (n = 31)	Filaria-negative (n = 31)
Change in overall filarial status	3	11
Loss of Wb mf positivity	2/2 (100%)	NA
Loss of Cag positivity	1/11 (9%)	NA
Loss of Mp mf positivity	5/20 (25%)	NA
Gain of Wb mf positivity	0	0
Gain of Cag positivity	5/20 (25%)	9/31 (29%)
Gain of Mp positivity	4/11 (36%)	4/31 (13%)

### Clinical malaria

**Figure 2:**
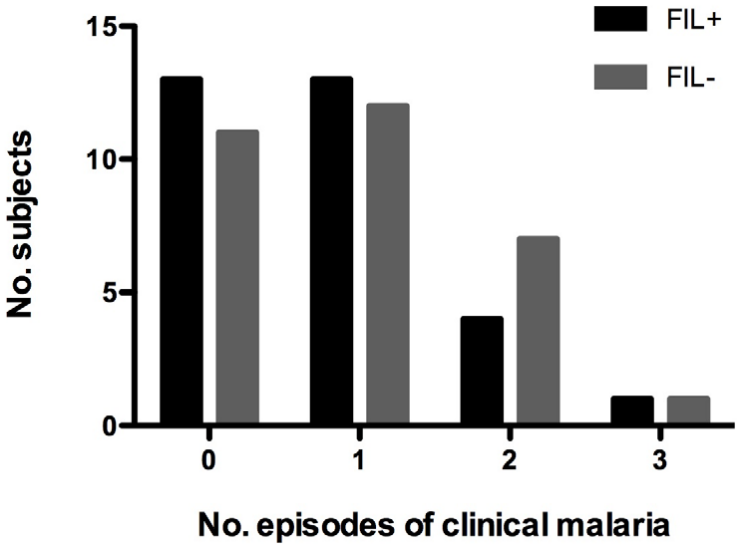
Incidence of clinical malaria. The bars represent the number of FIL+ (black bars) and FIL− (gray bars) subjects who experienced 0, 1, 2 or 3 episodes of clinical malaria during the first transmission season.

**Figure 3:**
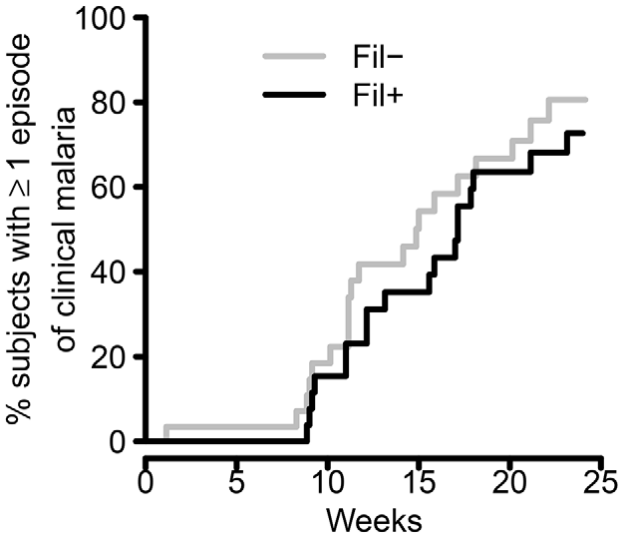
Time to first episode of clinical malaria. The cumulative % of FIL+ (black line) and FIL− (gray line) subjects who had experienced at least one episode of malaria is shown for each week of the first transmission season (estimated by Kaplan-Meier method to account for subjects who withdrew from the study).

**Figure 4:**
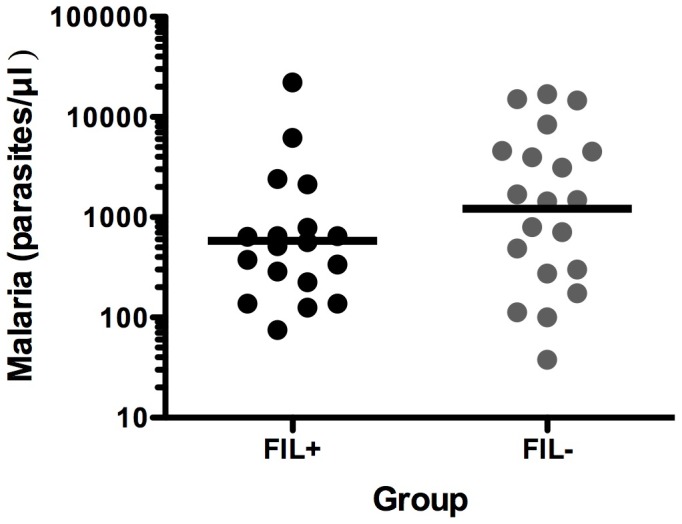
Parasitemia at the time of the first episode of clinical malaria. The symbols represent individual values for FIL+ (black circles) and FIL− (gray circles) subjects. The horizontal lines represent the GM values for the groups.

Although not all subjects were followed for the full 24 weeks season, 51.3 person-seasons of data were available for analysis. During the malaria transmission season, 38 (61%) subjects developed clinical malaria. There was no significant difference in the rates of clinical malaria between the FIL+ and FIL− groups (Rate ratio [FIL+/FIL−] = 0.85; 95% CI 0.49, 1.46; p = 0.56; Figure 2). Similarly, the time to first episode of clinical malaria was comparable with a median time to first episode of 17 weeks in the FIL+ groups and 15 weeks in the FIL− group (Hazard Ratio = 0.77; 95% CI 0.41, 1.45; p = 0.42; Figure 3). GM parasitemia at the time of the first episode of clinical malaria was also comparable between FIL+ and FIL− subjects (584 parasites/µl and 1216 parasites/µl, respectively; p = 0.18; GM ratio = 0.48; 95% CI 0.16, 1.40; Figure 4).

Clinical signs and symptoms were similar between the two groups at the time of diagnosis of the first episode of clinical malaria (Table 3), and no subjects met WHO criteria for severe malaria during the study. Fever, as defined by temperature >37.8 degrees was documented in 7/18 FIL+ and 5/20 FIL− subjects during their first episode of clinical malaria (p = 0.49). Mean temperatures were also comparable between the two groups (37.7 vs. 37.4 degrees Celsius in FIL+ and FIL−, respectively; p = 0.15; difference in means = 0.34; 95% CI 0.13, 0.81).

**Table 3:** Clinical signs and symptoms during first episode of clinical malaria.

	Filaria-positive (n = 18)	Filaria-negative (n = 20)
Temperature >37.8 degrees C	7 (39%)	5 (33%)
Geo Mean temperature (range)	37.7 (36.6–39.2)	37.4 (36.5–39)
Median Pulse (range)	80 (62–124)	81 (64–128)
Jaundice	0	0
Pallor	0	1 (5%)
Chills	10 (56%)	5 (25%)
Headache	8 (44%)	9 (45%)
Respiratory symptoms	2 (11%)	1 (5%)
Abdominal pain	4 (22%)	5 (25%)
Vomiting	2 (11%)	1 (5%)
Diarrhea	0	0
Myalgias	0	0

Note: There were no significant difference between the two cohorts for any of the clinical parameters studied.

**Figure 5:**
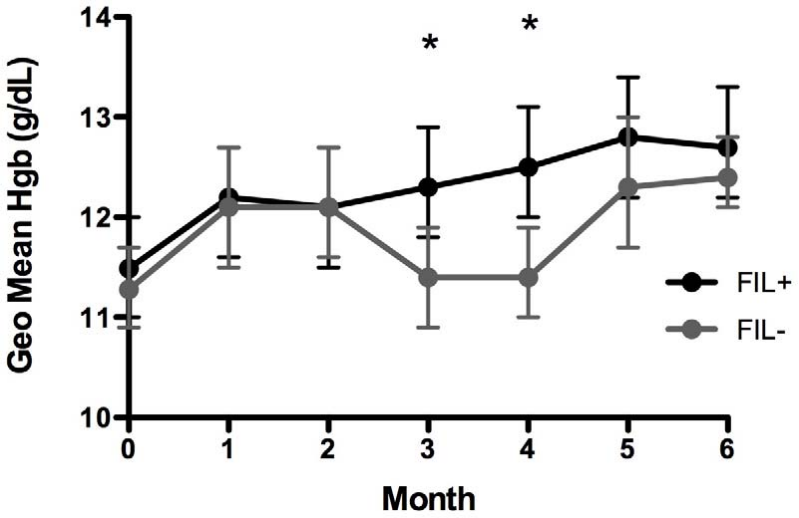
Hgb levels during monthly asymptomatic visits. GM Hgb with 95% confidence intervals are show for FIL+ (black) and FIL− (gray) subjects over time. *p<0.05, Mann-Whitney.

Hgb levels were measured monthly throughout the malaria transmission season to provide an indirect measure of chronic malaria morbidity (Figure 5). Values obtained during monthly visits at which clinical malaria was diagnosed were excluded from the analysis. GM Hgb levels were similar between the FIL+ and FIL− subjects at baseline (11.5 g/dL vs. 11.3 g/dL, respectively) and rose significantly in both groups at month 1 (11.5 to 12.2 g/dL in FIL+ and 11.3 to 12.1 g/dL in FIL−; p<0.01, Wilcoxon signed rank test). GM Hgb levels continued to slowly rise over the course of the transmission season in the FIL+ group, but decreased in the FIL− subjects during months 3 and 4 at the height of the malaria transmission season (from 12.1 to 11.4 g/dL; Figure 5) and were significantly lower than the GM Hgb values for the FIL+ group. Of note, the prevalence of asymptomatic malaria parasitemia was similar between the two groups at baseline (Table 1) and remained comparable throughout the transmission season (data not shown).

### Immune response to acute malaria infection

**Figure 6:**
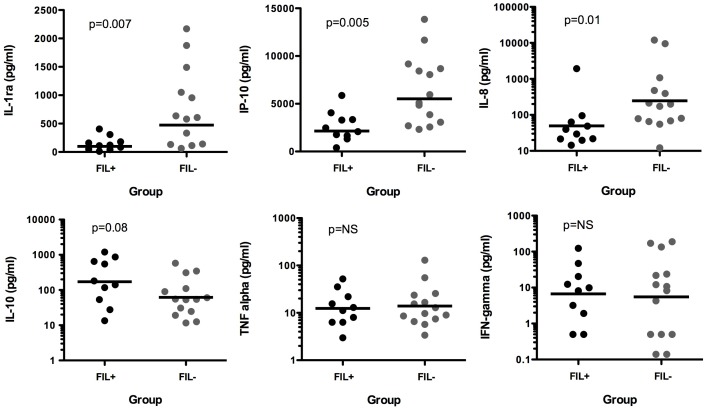
Plasma cytokine and chemokine levels at the time of acute malaria. The symbols represent individual values for FIL+ (black circles) and FIL− (gray circles) subjects. The horizontal lines represent the GM values for the groups.

Plasma levels of cytokines and chemokines previously reported to be associated with the severity of acute malaria were measured at the time of presentation with clinical malaria in FIL+ (n = 10) and FIL− (n = 14) subjects. Among the analytes measured, GM levels of IL-1ra, IP-10 and IL-8 were all significantly decreased in FIL+ subjects (99, 2145 and 49 pg/ml, respectively) as compared to FIL− subjects (474, 5522 and 247 pg/ml, respectively; Figure 6). In contrast, GM plasma levels of IL-10 were increased in FIL+ subjects (172 pg/ml) as compared to FIL− subjects (62 pg/ml), although this difference did not reach statistical significance (p = 0.08). Plasma levels of IFNγ, IL-1α, IL-1β, IL-6, IL-12p70 and TNFα were comparable between the two groups.

**Figure 7:**
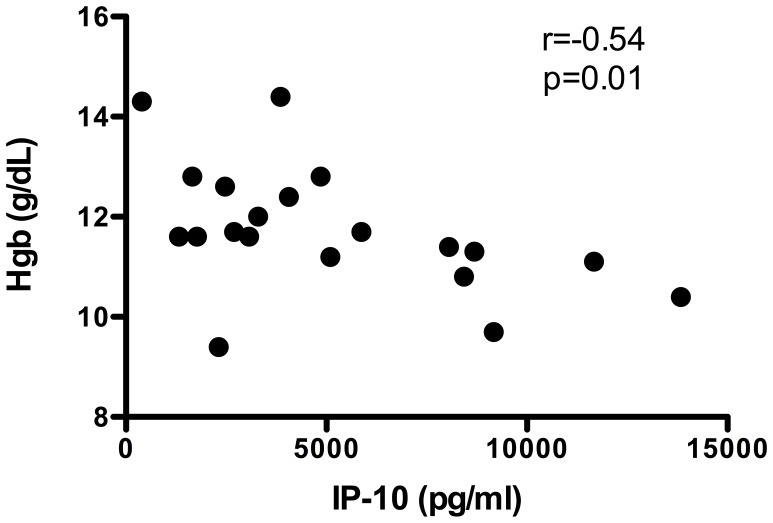
Negative correlation between serum levels of IP-10 during acute malaria and Hgb levels at the peak of malaria transmission. The symbols represent the values for individual study subjects.

In keeping with prior reports of an association between plasma levels of IP-10 and malarial anemia, plasma IP-10 levels measured at the time of acute clinical malaria were inversely correlated with Hgb levels at the peak of malaria transmission (r = −0.54, 95% CI −0,79, −0.11; p = 0.01; Figure 7). A negative correlation was also observed with the next measured Hgb level after the episode of acute malaria (r = −0.62, 95% CI −0.86, −0.16; p = 0.01; data not shown).

## Discussion

Despite considerable data supporting an effect of chronic helminth infection on immune responses to malaria parasites, longitudinal studies examining the incidence and severity of clinical malaria in helminth-infected and –uninfected individuals have been few. In the present study, 62 children and young adults with (FIL+) and without (FIL−) chronic filarial infection were matched for age, gender and hemoglobin type and followed longitudinally through a malaria transmission season. No significant differences were detected between FIL+ and FIL− subjects with respect to the incidence of clinical malaria, time to first episode of clinical malaria, number of episodes of clinical malaria during the transmission season, parasitemia at first episode, or clinical signs and symptoms at first episode. A prospective study of similar design that examined the effects of coinfection with schistosomiasis on clinical malaria in 338 Malian children aged 4–14 years of age demonstrated a significant increase in the time to first episode of clinical malaria by 10–14 days in coinfected children (p = 0.04), but only in the 4–8 year old age group [21]. Coinfected children, aged 4–8 years, also experienced slightly fewer episodes of clinical malaria (1.55 vs. 1.81 episodes, p = 0.03) during the transmission season. No differences were observed in the incidence of clinical malaria, number of episodes during the transmission season or parasitemia during an episode. Taken together, these two studies suggest that the effect of chronic helminth infection on the incidence and severity of acute clinical malaria is likely small and limited to younger age groups.

Despite the lack of an observable effect on the frequency or severity of clinical malaria, chronic filarial infection appeared to protect against the development of anemia during the height of the malaria transmission season (Figure 5). This is the exact opposite of what has been reported in the setting of hookworm infection [22], where hemoglobin levels were found to be significantly decreased in coinfected subjects compared to those with either hookworm or malaria infection alone. In the latter study, a protective effect of helminth infection on hemoglobin levels may have been outweighed by gastrointestinal blood loss in the hookworm-infected subjects. Although the mechanism by which chronic helminth infection might protect against anemia remains to be elucidated, there is evidence to suggest that pro-inflammatory cytokines play an important role in mediating malarial anemia [23].

Prior studies from our group and others have demonstrated increased plasma levels of IL-10, as well as increases in regulatory T cell frequency and function, in children and young adults with helminth infection [4], [16], [24], [25]. More importantly, most studies to date have demonstrated increased production of IL-10 and/or decreased production of inflammatory cytokines in helminth-infected individuals in response to malarial antigen stimulation in vitro [4], [26], [27]. In the present study, we examined serum cytokines at the time of acute malaria and found a similar pattern in vivo with a decrease in cytokines/chemokines associated with severe malaria (IL-1ra, IP-10 and IL-8) and an increase, albeit not statistically significant, in IL-10. Interestingly, these results are the opposite of what has been reported in similarly conducted studies of coinfection with schistosomiasis and malaria in Mali, in which coinfected children (4–14 years of age) had increased plasma IL-10 levels at baseline but a blunted response in the setting of malaria infection as compared to children without schistosomiasis [25]. The explanation for this discordance is unclear, but may relate to differences in the age groups of the subjects or characteristics of the infecting helminths studied.

The association between high plasma IP-10 levels and more severe malarial anemia has been reported previously [23], [28], and was confirmed in the present study (Figure 7). Of note, in a recent study conducted in Mali, the Fulani, an ethnic group known to be less susceptible to clinical malaria than other ethnic groups, were shown to have significantly higher plasma levels of IP-10 at baseline than Dogon children living in the same area, but no significant change in IP-10 levels in the setting of malaria infection [29], suggesting that the change in IP-10 levels may be a more important determinant of malarial anemia than baseline levels. In the present study, FIL+ subjects were found to have decreased plasma levels of IP-10 compared to FIL− subjects at the time of acute clinical malaria. Although plasma levels of IP-10 prior to the malaria transmission season were not available for the present study cohort, data from a cross-sectional study of 38 FIL+ and FIL− subjects in the same village suggest that the magnitude of the increase in plasma IP-10 levels in the setting of acute malaria infection was likely to have been greater in the FIL− cohort [4]. Furthermore, as demonstrated in the prior study, this blunting of the inflammatory response to malarial antigen in FIL+ subjects was most likely due to increased IL-10 [4].

A major anticipated limitation of the present study was the relatively small sample size due to the difficulty in recruiting FIL+ subjects in the younger age groups. Consequently, in order to maximize the possibility of detecting significant differences between the FIL+ and FIL− groups, a matched design was selected. Potential confounding variables, such as baseline malaria parasitemia, intestinal helminth infection, G6PD deficiency, bednet use and location of residence, were also similar between the two groups. Although subjects were not tested for HIV infection, the prevalence of HIV is extremely low in rural Mali and no subjects with clinical evidence of immunodeficiency were identified in the screening. Additional factors that may have diminished differences between the FIL+ and FIL− groups include lasting effects of intestinal helminth infection following treatment, decreased severity of malaria infection due to earlier intervention as a result of the presence of health care personnel in the village (as has been seen in other studies) and the possibility that some FIL− subjects may have had occult (microfilaria-negative) Mp infection or have acquired filariasis during the course of the study. In fact, 8 FIL− subjects became CAg-positive during the study and 4 became microfilaria-positive for Mp. Although these subjects were likely infected at the start of the study, they were considered FIL− for the purposes of the study (intent-to-treat analysis). In view of these considerations, it is likely that small (clinically insignificant) differences in responses between the FIL+ and FIL− groups were missed.

In summary, although pre-existent filarial infection attenuates cytokine/chemokine responses known to be associated with severe malaria, it appears to have little effect on susceptibility to or severity of acute malaria infection in children and young adults living in a malaria-endemic area with seasonal transmission. The apparent protective effect of filarial infection on decreased hemoglobin levels at the height of malaria transmission is intriguing and warrants further study in a larger cohort.
